# Impact of COVID-19 on Cancer-Related Care in the United States: An Overview

**DOI:** 10.3390/curroncol30010053

**Published:** 2023-01-04

**Authors:** Iktej Singh Jabbal, Saad Sabbagh, Barbara Dominguez, Mira Itani, Mohamed Mohanna, Thomas Samuel, Zeina Nahleh

**Affiliations:** Department of Hematology-Oncology, Maroone Cancer Center, Cleveland Clinic Florida, Weston, FL 33331, USA

**Keywords:** coronavirus, cancer care, COVID-19 vaccines, early detection of cancer, cancer screening

## Abstract

COVID-19 impacted several health services, including cancer-related care. Its implications were significant due to the lapse in hospital resources, compounded by the delays stemming from the economic effects on patients’ jobs and medical coverage. Furthermore, reports suggesting an increased risk for morbidity and mortality from COVID-19 in patients with cancer and those on active cancer treatment caused additional fear and potential delays in seeking medical services. This review provides an overview of the pandemic’s impact on cancer care in the United States and suggests measures for tackling similar situations in the future.

## 1. Introduction

In late 2019, initial reports of a novel coronavirus (SARS-CoV-2) in a province of China yielded an outbreak of coronavirus disease (COVID-19) that rapidly spread around the globe [1]. Little more than a month following the outbreak, the World Health Organization designated the spread as a “public health emergency of concern” and, ultimately, a pandemic [2]. At the time of drafting this review article, over 640 million cases were reported worldwide, with over 98.5 million of those cases originating in the United States (U.S.) alone [1]. The mortality rate of the disease was thought to be 2–5% in the general population. However, patients who were older or had underlying diseases, such as hypertension, diabetes, and chronic obstructive pulmonary disease, were considered high risk with a mortality rate of >50% [3,4].

The impact of the SARS-CoV-2 pandemic has been visible socially and economically. Medical resources and staff have been mobilized to accommodate the large, unanticipated influx of COVID-19-related hospitalizations. Due to this reallocation of resources and strict social sanctions implemented to limit the spread of the virus, access to care was disrupted for those with chronic health conditions [5]. The impact on cancer-related care, from diagnosis to palliation, was multifaceted, compounded by a lapse in hospital resources and the overall economic effects on the community, including patients’ health insurance and medical coverage [6].

This review article attempts to analyze the impact of the pandemic on cancer-related care and access to cancer services. Here, we suggest measures to consider to tackle similar situations in the future that may potentially impact the delivery of cancer-related care.

## 2. Delays in Cancer Screening

Screening and early symptom-based cancer detection, such as screening mammograms, colonoscopies, or pap smears, are essential to offer a prospective diagnosis of cancers at a stage when timely management can be curative or prolong survival. Unfortunately, during the early phases of the pandemic, government-mandated shutdowns, insufficiencies of healthcare organizations, and generalized anxiety and fear in the public’s minds led to a massive avoidance of many non-emergent hospital visits, including cancer screenings. This eventually led to a reduction in all outpatient elective activities, with cancer screenings being significantly underutilized. Additionally, many facilities had decreased elective visits and access to non-emergency procedures to increase their ability to manage patients with COVID-19 and its complications. As such, standard cancer screenings, such as breast cancer screenings, dropped by around 90% and colorectal cancer screenings by about 85% through May 2020 [7]. Furthermore, many cancer organizations, including the American Society of Clinical Oncology (ASCO) and the American College of Chest Physicians, similarly recommended delaying screenings such as mammograms, colonoscopies, and surveillance for lung cancer [8,9]. Some experts also proposed delaying cervical and prostate cancer screenings given that they were slow-growing malignancies and that outcomes would likely be minimally affected [10].

As a result of this general trend, a significant drop in the weekly rate of routine cancer screenings was noted early towards the start of the pandemic, as reported by a study conducted by Epic Research Health Network (ERHN) based on a dataset of 107 million patients [11]. Standard cancer screenings declined rapidly, with those for breast cancer seen to decline by 94%, colon cancer screenings by 86%, and cervical cancer screenings by almost 94%. While a follow-up study by ERHN in July 2020 showed a rise in screening rates, these were still seen to remain 29–36% lower than the pre-COVID-19 levels. These numbers have continued to rise due to the reinvigorated efforts of many organizations. For example, breast cancer screenings in March 2021 rose to levels that were 13% below historical averages, while colon cancer and cervical cancer screenings rose to around 25% below the average values. While a similar rebound in cancer screening rates was reported by another study [12], these missed screenings continue to raise the concern of an increase in later-stage cancer diagnoses with potentially poorer prognoses. Data from the Washington State Surveillance, Epidemiology, and End Results (SEER) registry revealed an increased incidence of stage IV and a decrease in stage I cases of colorectal cancer during both the early and late phases of the pandemic, highlighting the short-term and potential long-term effects of the interruption of screening tests [13]. While our findings suggest that the gap is decreasing, continued diligence to raise awareness and promote advocacy by partnering with cancer societies and national oncology organizations would be needed to turn the tide and regain the momentum to improve cancer screening services. Plus, increased efforts are recommended to identify the population with interrupted screening schedules to halt the potential progression of occult disease. Future implications would be to consider continuing cancer screening services uninterrupted by healthcare facilities and tackling screening tests as essential services during similar situations in the future, given the far-reaching implications that these interruptions might cause.

## 3. Decrease in New Cancer Diagnoses and Rise in Cancer-Related Deaths

As noted above, efforts to control the pandemic and prioritize resources to tackle the new challenges imposed by COVID-19 translated into decreasing rates of cancer screening and, consequently, cancer diagnoses as the pandemic progressed by around 65% by April 2020 [5]. For example, the incidence of new lung cancer diagnoses dropped by 46.8%, and new melanoma diagnoses dropped by 67.1% in April 2020 compared with 2019, as noted in one study [5]. Another report evaluating patient encounters between January–April 2019 and January–April 2020 across multiple clinics across the U.S. similarly reported a substantial decline in the number of patients with cancer encounters [5], including a reduction in the incidence of lung cancer (–46.8%), breast cancer (–50.5%), prostate cancer (–46.8%), colorectal cancer (–54.2%), hematologic cancers (–54.2%), and melanoma (–67.1%) cases between April 2019 and 2020 [5]. What was also notable during this period was that several centers modified their cancer treatment delivery and either switched to the oral mode of administration when feasible and/or to less aggressive treatment regimens to reduce the need for visits to facilities [14].

The significant change in cancer care during the pandemic raised obvious concerns that progress made in cancer advances over the years would be significantly compromised for many years to come. Pre-pandemic cancer statistics related to outcomes in the U.S. had shown continuous improvement, including a 25% drop in cancer mortality over the last 25 years [15]. Nevertheless, this improvement in survival was offset during 2020, as a study led by researchers at the American Cancer Society estimated a 3.2% increase in cancer-related death in the US when comparing data between every month of 2019 and 2020 [16]. It is feared that the decrease in new cancer detection rates due to COVID-19 during the pandemic would potentially jeopardize the progress achieved and lead to increasing the number of late-stage cancers and decreasing cancer survival in the U.S. [17]. It has indeed been projected that in the U.S., shutdowns related to COVID-19 would result in a projected delay of more than 22 million screening tests for cancer and a 20% reduction in oncology visits [18]. The National Cancer Institute has estimated a 1% increase in breast and colorectal cancer deaths over the next 10 years, the equivalent of approximately 10,000 excess deaths due to the pandemic’s impact on screening and treatment [19]. These findings were replicable in other countries, where a similar worrisome trend has been observed. In the United Kingdom, an analysis using the National Health Service (N.H.S.) data on cancer diagnoses estimated a 7.9% to 9.5% increase in deaths from breast cancer due to later stages at diagnosis [20]. Similarly, a study in Canada estimated a 23% decrease in the number of new cancer diagnoses (ratio = 0.77, 95% CI 0.67–0.87) and a 21% decrease in the number of pathology reports (ratio = 0.79, 95% CI 0.70–0.88) at the start of the pandemic. The same study also reported a 43% reduction in surgical resections (ratio = 0.57, 95% CI 0.45–0.70) in April 2020 [21]. Cancer-directed surgery was globally impacted to a greater degree as surgical resources and hospital rooms for patient recovery were redirected to address COVID-19 needs [21].

When comparing the mean percentage change in newly started non-COVID-19 clinical trials for every month between 2019 and 2020, there was a 7.5% reduction, with a maximum of 57.3% reduction observed during April. This study was conducted using data from clinicaltrial.gov, the largest clinical trial database, and encompasses data on trials from 209 countries, including the U.S. An inversely proportional relation was found between the activity of clinical trials and the number of COVID-19 patients [22]. Furthermore, the deterioration of patients’ overall health and inability to be recruited or involved in clinical trials posed an additional worse prognostic burden on patients with malignancy.

It would be difficult to speculate on the best approach for cancer treatment under similar circumstances, as it would be heavily dependent on the resources available at the time. Still, it would be reasonable to suggest that every effort should be made to continue cancer surgeries and treatment with minimal interruptions and coordinate care and resources among facilities to ensure continuity of cancer care should future challenges arise that are similar to what was seen during the pandemic circumstances.

## 4. COVID-19 Vaccines in Patients with Cancer

Since identifying SARS-CoV-2 as the causative agent of COVID-19, vaccines with very high efficacy were developed and deployed with remarkable speed. Several independent trials demonstrated high efficacy against symptomatic disease. Despite being a vulnerable population, patients with cancer were underrepresented in the early pivotal trials of the COVID-19 vaccines. Although a few small studies conducted in the U.S. and other countries indicated that the messenger ribonucleic acid (mRNA) vaccines were safe for patients under active cancer treatment, the safety and efficacy of the vaccines in patients with cancer remain understudied [23,24,25,26]. Despite the limitations, guidelines in the U.S. and Europe, including those of ASCO and the European Society for Medical Oncology (ESMO), have strongly supported the vaccination of all patients with cancer, including those under active treatment. Nevertheless, the attitudes of patients with cancer hindered compliance with vaccination efforts largely due to concerns about vaccine side effects, disbelief in their efficacy, and underestimation of the seriousness of contracting the infection [27].

It is well recognized that patients on active cancer therapy, those diagnosed with hematologic malignancies and solid tumors, or post-organ transplant patients may not mount the desired immune response to the vaccines as their non-immunocompromised counterparts would [28]. As such, several recent studies have pointed out higher seronegativity in patients with cancer compared to the rest of the population [25,29,30,31]. Ehmsen et al. assessed anti-SARS-CoV-2 spike immunoglobulin G (IgG) antibody and T-cell responses in patients with cancer who received the Pfizer-BioNTech vaccine or the Moderna vaccine [25]. The study included patients with solid tumors and hematologic malignancies and found a 93% seropositivity rate in patients with solid tumors and 66% in those with hematologic malignancies (*p* = 0.004). Patients receiving anti-CD20 therapy, Bruton’s tyrosine kinase (B.T.K.) inhibitor therapy, or chemotherapy were found more likely to be seronegative. Among patients with a solid tumor, 46% had a T-cell response, out of whom 76% had both CD4+ and CD8+ T-cell responses. Among patients with hematologic malignancies, 45% had a T-cell response, 81% of whom had both CD4+ and CD8+ T-cell responses. The number of detectable antibodies dropped from 93% at 36 days after immunization to 86% at three months after. Addeo et al. [29] reported seroconversion rates and median antibody titers being significantly lower in hematologic malignancy groups than in solid tumor groups (77% vs. 98%, *p* = 0.002). Patients who received cytotoxic chemotherapy (*p* = 0.019) or monoclonal antibody therapy (*p* = 0.029) within six months before the first vaccine dose had lower antibody levels than those not on active treatment.

## 5. COVID-19 Infection in Patients on Active Cancer Treatment

Patients with cancer were reported to have an increased risk for morbidity and mortality from COVID-19, and active treatment was suggested to increase these risks further [32]. However, timely care and access to planned life-saving treatment are crucial for this vulnerable population [33]. Reconciling these challenges became a priority during the early days of the pandemic, and several studies were initiated to better evaluate the optimal approach for patients with cancer. For example, a study suggested that all-cause mortality and the need for intensive care unit (I.C.U.) admission are higher in cancer patients versus the general population [34], while another indicated that immunocompromised states make cancer patients more susceptible to infection [35]. Another study analyzed 218 individuals with COVID-19 and cancer and reported a significant increase in COVID-19-related mortality in patients with cancer compared to their counterparts [36]. Similarly, increased mortality and morbidity were observed in another analysis, which included 928 patients with COVID-19 and cancer [37]. Efforts to gather additional data on the effect of COVID-19 on the cancer care continuum, with several key organizations, such as the “COVID-19 and Cancer Consortium” and ASCO, are working towards guiding optimal cancer care during the pandemic [38,39].

Contrary to the potentially increased expected adverse effects noted in cancer patients with COVID-19 exposure, the findings in a recent study by Foote et al. are encouraging [40]. The study reported a statistically significant lower incidence of SARS-CoV-2 infection in patients who received potential angiotensin-converting enzyme-2 lowering antineoplastic compared to those on other therapies. Notable among these drugs were drugs that inhibit the mechanistic target of rapamycin (mTOR)/Phosphoinositide 3-kinase (PI3K) inhibitors (everolimus, temsirolimus, and alpelisib) and antimetabolites (gemcitabine and decitabine) [40]. Antimetabolites have previously been found to have an association with lower COVID-19-related mortality in patients with cancer [41]. While the authors of this study agreed that the small sample size was a limitation, the results were nonetheless encouraging. This also paves the way for future research exploring the molecular mechanisms underlying anti-SARS-CoV-2 activity for potential ACE-2-lowering antineoplastic agents.

## 6. A Call for Action

Despite all the challenges imposed by the pandemic on healthcare systems, this pandemic has created processes for the rapid implementation of safety protocols and measures, such as telemedicine consultations, to manage and overcome the barriers to patient care. Telemedicine has witnessed dramatic growth during the pandemic era. It provided patients the ability to maintain cancer care and follow-up while avoiding exposure to threatening pathogens at the hospital or in public. Plus, telemedicine facilitated preexisting physical and demographic barriers imposed on cancer patients. Due to its novelty, additional measures are consistently evolving to improve the care provided through telemedicine and will remain in place to help future global disease threats.

Initiatives such as training a more significant number of providers to screen patients and increasing the workforce at cancer centers to capture more patients would be desirable in this regard. The development of specialized screening centers and dedicated outpatient department infusion centers can be a solution for maintaining continuity of care with minimal exposure. Cancer care is essential, and future measures to minimize interruptions of cancer services will be needed during future challenges. More research is required to evaluate creative options and replace the dependence on in-person screenings, such as developing more individualized screening approaches and home-based screening tests.

Future clinical trials studying appropriate schedules for vaccination in patients with cancer to achieve adequate immunity should be developed. In addition, prospective studies examining the responses of specific cancer types to vaccination would help direct cancer subpopulations that would receive the maximum benefit from vaccination. Furthermore, studying the interactions of specific chemotherapy regimens with vaccines would guide physicians in choosing the right drugs for these patients. Moreover, assessing the need for social isolation and other strategies for mitigating the risk of contracting COVID-19 by immunosuppressed patients, developing best practices, and designing and rapidly conducting clinical studies focused on passive immunization strategies would also be helpful.

The COVID-19 pandemic has been associated with a sharp decline in cancer screening and detection rates, translating into patients being diagnosed at later stages of the disease and potentially compromising survival outcomes. Moreover, there was a serious decrease in the rate of admission to elective and emergency services during the pandemic period. While this situation caused serious complications in patients requiring emergent care, it led to delays and stage skipping in cancer patients. The specific impact of delayed cancer-related care and delayed follow-up appointments during the pandemic will not likely be evident immediately [42,43,44]. However, ongoing prospective studies will assess the long-term effects of the COVID-19 pandemic on patients with cancer. For example, the National Cancer Institute (NCI) is currently studying 2000 patients undergoing cancer treatment who have also been infected with COVID-19 and will be followed for up to 2 years [45]. In addition, other longer-term prospective cohort studies, including the American Cancer Society’s Cancer Prevention Study-3, have also collected information about COVID-19 to examine its effects on cancer outcomes [46].

## 7. Conclusions

While the effects of delays in cancer screening and diagnosis were significant during the pandemic, the entire medical community is now invited to collaborate on promoting advocacy to continue standard cancer screenings and compliance with early detection to reach the pre-pandemic cancer screening levels and salvage the progress in cancer outcomes achieved over the last several decades. The implications of the COVID-19 pandemic did not only affect cancer care. They have extended to compromise the care of patients with chronic diseases. Uncontrolled hypertension, asthma plus COPD exacerbations, renal injury, and a compounded risk of thrombotic events were more frequently observed in patients with active infection [47]. These implications reaffirm the necessity of establishing preemptive and regulatory measures to maintain the comprehensive care of all patients and control the impacts of similar disastrous crises in the future.
